# Physiologic Recovery After Smoking Cessation: A Systematic Review of Inflammatory and Cardiovascular Biomarker Changes

**DOI:** 10.7759/cureus.112444

**Published:** 2026-07-11

**Authors:** Ahmad Mohammad, Uday Shree Akkala Shetty, Roland Asiwe, Hafiz H Zubair, Zaeem Ahmad, Aryan Raj, Daniel E Cook, Ahmad Sattar

**Affiliations:** 1 Internal Medicine, Hurley Medical Center, Flint, USA; 2 Internal Medicine, Southern Regional Medical Center, Riverdale, USA; 3 Internal Medicine, Leeds Teaching Hospitals NHS Trust, Leeds, GBR; 4 Internal Medicine, Pilgrim Hospital Boston, Boston, GBR; 5 Internal Medicine, Nottingham University Hospitals NHS Trust, Nottingham, GBR; 6 Internal Medicine, Mayo Hospital, Lahore, PAK; 7 Internal Medicine, Shaheed Mohtarma Benazir Bhutto Medical University, Larkana, PAK; 8 Internal Medicine, Avalon University School of Medicine, Willemstad, CUW; 9 Internal Medicine, Allama Iqbal Medical College, Lahore, PAK

**Keywords:** biomarkers, cardiovascular risk, c-reactive protein, endothelial dysfunction, f2-isoprostanes, fibrinogen, inflammation, lipid profile, oxidative stress, smoking cessation

## Abstract

Cigarette smoking remains a major modifiable contributor to cardiovascular morbidity and mortality through mechanisms involving systemic inflammation, oxidative stress, endothelial dysfunction, dyslipidemia, and hematologic abnormalities. Although the long-term cardiovascular benefits of smoking cessation are well-established, the temporal pattern of biologic recovery following cessation remains incompletely understood. This systematic review synthesized current evidence regarding the reversal of cardiovascular risk biomarkers after smoking cessation, with emphasis on the timing and magnitude of biomarker normalization. A comprehensive search of PubMed, Embase, Scopus, and Cochrane CENTRAL identified studies published between January 1990 and May 2024 evaluating biomarker changes following smoking abstinence. Eligible studies included randomized controlled trials, prospective clinical studies, and observational investigations involving adults who achieved smoking abstinence for at least seven days. Risk of bias was assessed using the Cochrane Risk of Bias 2 (RoB 2) tool and the Risk of Bias in Nonrandomized Studies-of Interventions (ROBINS-I) instrument, and findings were synthesized narratively because of methodological heterogeneity. Ten studies comprising 2,750 participants met the inclusion criteria. Biomarker recovery followed a staged temporal pattern, with oxidative stress markers improving within one to two weeks, inflammatory biomarkers declining over approximately two to 12 weeks, and lipid and hematologic parameters improving more gradually over three to eight months. However, substantial heterogeneity in study design, cessation interventions, biomarker selection, and follow-up duration limited direct cross-study comparability. Adjunctive interventions, including antioxidant supplementation and exercise, appeared to enhance biomarker recovery in selected populations. Overall, smoking cessation was associated with progressive improvement in multiple cardiovascular risk pathways, supporting its critical role in cardiovascular prevention and highlighting the potential value of biomarker-guided approaches in future risk-reduction strategies.

## Introduction and background

Tobacco smoking remains one of the most important modifiable contributors to cardiovascular morbidity and mortality worldwide. Exposure to cigarette smoke promotes a broad spectrum of pathological processes, including oxidative stress, endothelial dysfunction, platelet activation, vascular inflammation, and adverse lipid remodeling, all of which contribute to the initiation and progression of atherosclerotic cardiovascular disease (CVD) [1]. Although smoking prevalence has declined in several high-income countries, smoking-related CVD continues to impose a substantial global burden, particularly in low- and middle-income regions where cessation support strategies may remain inconsistently implemented or inadequately accessible [2,3].

A substantial body of epidemiological evidence has established a strong association between smoking and major adverse cardiovascular outcomes, including myocardial infarction, stroke, peripheral arterial disease, and cardiovascular mortality [4]. At the biological level, these effects are mediated through alterations in inflammatory, oxidative, hematologic, and atherogenic pathways. Biomarkers such as high-sensitivity C-reactive protein (hs-CRP), interleukin-6 (IL-6), tumor necrosis factor-alpha (TNF-α), fibrinogen, homocysteine, oxidized low-density lipoprotein (oxLDL), and soluble intercellular adhesion molecule-1 (sICAM-1) have been implicated in smoking-related vascular injury and cardiovascular risk propagation. Beyond their prognostic significance, these biomarkers may also provide mechanistic insight into disease progression and represent potentially modifiable targets for cardiovascular risk reduction [5].

Smoking cessation has consistently been associated with meaningful reductions in long-term cardiovascular risk and mortality. However, the temporal dynamics and magnitude of biologic recovery following cessation remain incompletely characterized [6]. Existing evidence suggests that certain pathophysiological processes, particularly oxidative stress and endothelial dysfunction, may improve rapidly after smoking cessation, whereas normalization of inflammatory and metabolic biomarkers may occur more gradually or inconsistently across populations. Interpretation of the available literature is further complicated by heterogeneity in study design, patient characteristics, cessation strategies, biomarker selection, and follow-up duration, limiting the development of a unified understanding of post-cessation biomarker recovery [7,8].

Despite the clinical relevance of this topic, contemporary evidence synthesizing the temporal patterns of cardiovascular biomarker recovery following smoking cessation remains limited. A clearer understanding of the biologic trajectory of recovery after smoking abstinence may strengthen cardiovascular prevention strategies, improve patient counseling, and help contextualize the early physiologic benefits of cessation. Therefore, this systematic review aims to critically evaluate and synthesize current clinical evidence regarding the effects of smoking cessation on inflammatory, oxidative stress, lipid, and hematologic biomarkers associated with cardiovascular risk. By examining measurable physiologic changes across multiple biomarker domains, this review seeks to provide a clinically and mechanistically informed overview of cardiovascular recovery following smoking cessation.

## Review

Materials and methods

Study Design and Reporting Standards

This systematic review was conducted in accordance with the Preferred Reporting Items for Systematic Reviews and Meta-Analyses (PRISMA) 2020 guidelines [9]. The review protocol was developed in advance to ensure methodological transparency and rigor. The research question was structured using the population, intervention, comparison, and outcome (PICO) framework [10], which guided the definition of the review scope, eligibility criteria, and outcomes of interest.

PICO Framework

The population of interest included adult cigarette smokers, with or without underlying cardiovascular risk factors or established CVD. The intervention consisted of smoking cessation achieved through behavioral counseling, pharmacologic therapies, or combined approaches, with or without adjunctive interventions such as antioxidant supplementation or exercise. Comparison groups included continued smokers, baseline biomarker levels before cessation, or internal pre- and post-cessation comparisons. The primary outcomes were changes in cardiovascular risk biomarkers, including inflammatory markers (e.g., CRP, TNF-α, and IL-6), oxidative stress markers (e.g., F2-isoprostanes and malondialdehyde (MDA)), lipid parameters (e.g., low-density lipoprotein (LDL) and high-density lipoprotein (HDL)), and hematologic markers such as white blood cell (WBC) count and fibrinogen.

Eligibility Criteria

Studies were eligible for inclusion if they involved human participants aged 18 years or older and presented original clinical data from randomized or nonrandomized trials, observational cohorts, or prospective interventional studies. Studies must have examined the impact of smoking cessation, defined as abstinence for at least seven days, on at least one biomarker of interest. Only articles published in peer-reviewed journals and written in English between 1990 and 2024 were included. Exclusion criteria encompassed nonhuman studies, in vitro experiments, case reports, reviews, editorials, and letters without original data. Studies focused solely on smoking reduction without full cessation, or those involving dual tobacco product users without clear cessation endpoints, were also excluded.

Search Strategy

A comprehensive search of PubMed, Embase, Scopus, and Cochrane CENTRAL databases was conducted for studies published from January 1990 to May 2024. Search terms and Boolean combinations included phrases such as “smoking cessation,” “tobacco abstinence,” “quit smoking,” and keywords related to biomarkers, such as “inflammatory markers,” “oxidative stress markers,” “lipid profile,” and “cardiovascular risk.” Specific terms like CRP, fibrinogen, WBC, F2-isoprostanes, MDA, LDL, HDL, homocysteine, ICAM, VCAM, and platelet volume were also included. Additionally, reference lists of relevant studies were manually screened to identify any further eligible articles.

Study Selection Process

All retrieved titles and abstracts were screened independently by two reviewers. Full-text articles of potentially eligible studies were then reviewed in detail to assess eligibility based on the predefined inclusion and exclusion criteria. Any discrepancies in study selection were resolved through discussion or, when necessary, consultation with a third reviewer. The study selection process was documented in a PRISMA flow diagram, which illustrates the number of records identified, screened, excluded, and ultimately included in the review.

Data Extraction

A standardized data extraction form was developed and pilot-tested before formal data collection. Extracted variables included study authorship, year of publication, study design, population characteristics, smoking cessation intervention details, sample size, type and timing of biomarker measurements, and main outcomes. Two reviewers independently extracted data to minimize the risk of error or bias. Discrepancies were reconciled through discussion and review of the original articles.

Risk of Bias Assessment

The risk of bias in the included studies was assessed using the Risk of Bias in Nonrandomized Studies-of Interventions (ROBINS-I) [11] tool for nonrandomized studies and the Cochrane Risk of Bias 2.0 (RoB 2) tool [12] for randomized controlled trials. Each study was evaluated across domains such as confounding, participant selection, outcome measurement, and deviations from intended interventions. Studies were then categorized as having low, moderate, or high/serious risk of bias based on the overall assessment.

Data Synthesis

Due to the heterogeneity in study design, intervention strategies, biomarker types, and outcome measurements, a meta-analysis was not feasible. Instead, a narrative synthesis approach was adopted. Findings were grouped and analyzed by biomarker class, namely, inflammatory, oxidative stress, lipid/cardiometabolic, and hematological markers. The synthesis focused on identifying consistent trends, evaluating response timelines across biomarker types, and exploring modifying factors, such as body mass index (BMI), sex, and the presence of adjunctive interventions.

Results

Study Selection Process

**Figure 1:**
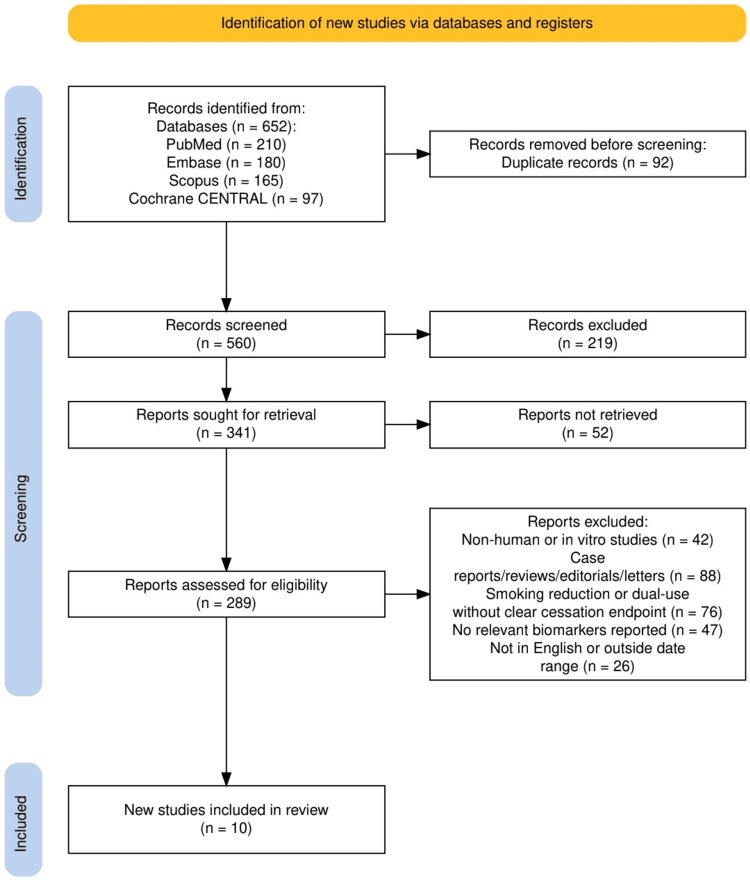
The PRISMA flow diagram represents the study selection process PRISMA: Preferred Reporting Items for Systematic Reviews and Meta-analyses

The study selection process is illustrated in Figure 1, adhering to the PRISMA 2020 flow diagram format. A total of 652 records were identified across four major databases: PubMed (n = 210), Embase (n = 180), Scopus (n = 165), and Cochrane CENTRAL (n = 97). After removing 92 duplicate records, 560 unique records were screened for eligibility. Of these, 219 were excluded based on title and abstract screening, and 341 full-text articles were sought for retrieval. However, 52 could not be retrieved, leaving 289 articles for full-text review. Subsequently, 42 nonhuman or in vitro studies, 88 case reports, reviews, editorials, or letters without original data, 76 studies focused solely on smoking reduction or dual tobacco use without a clear cessation endpoint, 47 studies that did not report relevant biomarkers, and 26 that were either not in English or published outside the designated date range (1990-2024) were excluded. Ultimately, 10 studies met all inclusion criteria and were included in the final qualitative synthesis.

Characteristics of the Selected Studies

Table 1 summarizes the characteristics of the 10 included studies. The evidence base comprised randomized trials, prospective clinical studies, and observational investigations involving populations ranging from healthy smokers to individuals with cardiovascular risk factors or established atherosclerotic disease. Interventions included smoking cessation support with nicotine replacement therapy, behavioral counseling, exercise or lifestyle modification, antioxidant supplementation, and, in one study, switching to a heated tobacco system. Biomarkers assessed included inflammatory markers, oxidative stress indicators, lipid parameters, endothelial function measures, and hematologic indices, with follow-up ranging from several days to more than one year. Overall, favorable biomarker changes were most consistently observed in oxidative stress and inflammatory domains, while lipid and hematologic improvements generally occurred over longer follow-up intervals. These findings were synthesized narratively because of substantial heterogeneity in study design, intervention type, biomarker selection, and follow-up duration.

**Table 1:** Characteristics of included studies evaluating biomarker changes following smoking cessation ApoA1: apolipoprotein A1; BMI: body mass index; CHD: coronary heart disease; CRP: C-reactive protein; CVD: cardiovascular disease; DBP: diastolic blood pressure; DART: Diet and Reinfarction Trial; FMD: flow-mediated dilation; HbA1c: hemoglobin A1c; HDL: high-density lipoprotein; HR: heart rate; hs-CRP: high-sensitivity C-reactive protein; ICAM-1: intercellular adhesion molecule-1; IL-6: interleukin-6; LDL: low-density lipoprotein; MDA: malondialdehyde; mTHS: menthol Tobacco Heating System; MPO: myeloperoxidase; NRT: nicotine replacement therapy; oxLDL: oxidized low-density lipoprotein; PGF2α: prostaglandin F2 alpha; RCT: randomized controlled trial; RBC: red blood cell count; SBP: systolic blood pressure; sTNFR-I: soluble tumor necrosis factor receptor I; sTNFR-II: soluble tumor necrosis factor receptor II; sVCAM-1: soluble vascular cell adhesion molecule-1; tcpO2: transcutaneous oxygen pressure; TNF-α: tumor necrosis factor alpha; VO₂max: maximal oxygen consumption; WBC: white blood cell count

Study (author, year)	Study design	Population characteristics	Smoking cessation intervention	Comparison group	Sample size	Biomarkers measured	Measurement timepoints	Main findings
Korhonen et al., 2011 [13]	RCT (2×2 factorial design)	Sedentary female smokers aged 19-55 years, Greater Boston area	Nicotine patch + brief behavioral counseling + moderate-intensity exercise for 15 weeks	No separate nonintervention group; comparisons among abstainers vs. relapsers; home vs. facility exercise	130 completed (all received intervention with stratified exercise setting)	WBC, RBC, total cholesterol, HDL, total cholesterol:HDL ratio, HR, HbA1c, BMI, VO₂max	Baseline, 12 weeks post-cessation	↓ WBC, ↓ RBC, ↑ VO₂max (all); ↓ total cholesterol & cholesterol:HDL ratio, ↓ HR (abstainers); ↑ BMI and HbA1c (all)
Stein et al., 2002 [14]	Clinical trial	Healthy adult smokers (mean 35.9 ± 6.4 cigarettes/day)	Nicotine inhaler + individualized cessation counseling	Continued smokers and smoking reducers (4-8 cigarettes/day)	51 total participants	Plasma homocysteine	Baseline, post-intervention (duration not specified)	Smoking cessation reduced homocysteine by 11.6%; no change in reducers or continued smokers
Reichert et al., 2009 [15]	Pilot clinical trial	Women at risk for CVD enrolled in a smoking cessation program	Structured smoking cessation program with 4 clinic visits over 6-7 weeks	No formal control group; self-comparison across visits	46 women	CRP, IL-6, TNF, sTNFR-I, sTNFR-II, sVCAM-1	Visit 1 (baseline while smoking), visits 2-4 (post-cessation, 6-7 weeks)	Significant ↓ in TNF, sTNFR-I/II, and sVCAM-1; nonsignificant ↓ in IL-6 and CRP
Haustein et al., 2002 [16]	Open, parallel-group clinical trial	197 male smokers (aged 25-45, >20 cigarettes/day)	12-week NRT program with follow-up at 26 weeks	33 continued smokers (control group)	197 (164 intervention, 33 control)	Fibrinogen, plasma viscosity, erythrocyte deformability, tcpO2, hematocrit, WBC count	Baseline, 4, 8, 12, and 26 weeks	Significant ↓ in fibrinogen, ↑ in tcpO2 and capillary flow in abstainers; WBC and hematocrit also decreased. NRT did not negate improvements
Haziza et al., 2020 [17]	Randomized controlled clinical trial (3-arm parallel group)	160 healthy adult U.S. smokers	Smoking abstinence and switching to menthol Tobacco Heating System (mTHS) 2.2 for 90 days	Menthol cigarette users and abstainers	160 (2:1:1 ratio)	hs-CRP, total/HDL/LDL cholesterol, glucose, 11-dehydrothromboxane B2, fibrinogen, ICAM-1, homocysteine, 8-epi-prostaglandin F2α, SBP/DBP, ApoA1, triglycerides	Baseline, Day 5, Day 90	Switching to mTHS improved multiple biomarkers of inflammation, oxidative stress, lipid profile, and endothelial function. Effects were comparable to smoking cessation, especially in normal weight individuals
Tonstad et al., 2005 [18]	Parallel-group randomized controlled trial	172 young adults (18-39 yrs) with ≥1 lipid abnormality and family history of premature CHD; 40% were smokers	Intensive lifestyle intervention (smoking cessation support + dietary counseling)	General lifestyle advice	172	LDL, oxidized LDL, E-selectin, ICAM-1	Baseline, ~8 months	Intervention group reduced LDL, oxidized LDL, and E-selectin significantly. ICAM-1 decreased in quitters versus continued smokers. Smoking cessation and dietary changes showed favorable cardiometabolic effects
Chehne et al., 2002 [19]	Prospective observational clinical trial	71 patients (32-67 yrs) with clinically manifest atherosclerosis + 8 hypercholesterolemic patients without atherosclerosis	Smoking cessation (monitored weekly); some participants restarted smoking	Internal comparison (pre/post cessation and resumption)	79 (71 with atherosclerosis, 8 without)	8-epi-prostaglandin F2α (in plasma, serum, urine)	Baseline, 1–2 weeks after cessation, and after relapse	Significant decrease in oxidative stress marker (8-epi-PGF2α) after 1-2 weeks of cessation. Marker levels rebounded to baseline within 1 week of resuming smoking. Shows rapid and reversible oxidative injury pattern linked to smoking
Mah et al., 2013 [20]	Randomized, double-blind, placebo-controlled trial	30 healthy young smokers (mean age 22 ± 1 years)	Smoking cessation for 7 days + γ-tocopherol (500 mg/day)	Placebo group undergoing smoking cessation only	30 (γ-T group: 16; Placebo: 14)	FMD (vascular function), cotinine, γ-T, MDA, oxLDL, F2-isoprostanes, TNF-α, MPO	Baseline and after 7 days	Smoking cessation reduced cotinine and MDA in both groups. γ-T group showed enhanced FMD (+1.3%), and decreases in TNF-α and MPO, not seen in placebo. Antioxidant co-therapy added vascular benefit beyond cessation alone
Burr et al., 1992 [21]	Clinical trial using data from DART	1,755 men recently recovered from acute myocardial infarction	Post-MI smoking cessation (self-reported)	Continued smokers post-MI	1,755 (92 deaths)	Plasma fibrinogen, plasma viscosity, WBC count, hemoglobin, mean platelet volume	Baseline and 18-month follow-up	Mortality was lower in quitters (4.1%) than continued smokers (7.9%), associated with reduced fibrinogen. Hematological markers like fibrinogen and WBC were strong prognostic indicators. Smoking cessation reduced fibrinogen, improving prognosis
Dietrich et al., 2002 [22]	Randomized, double-blind, placebo-controlled trial	126 adult smokers (age range: 20-78 years; mean age: 46) stratified by BMI	Antioxidant supplementation (vitamin C alone vs. combination with vitamin E and alpha-lipoic acid) for 2 months	Placebo group	126	Plasma F(2)-isoprostanes (marker of lipid peroxidation)	Baseline and 2 months post-intervention	In smokers with high BMI, vitamin C alone significantly reduced F(2)-isoprostanes. The antioxidant mixture was not significantly better. No benefit was seen in the low BMI group. BMI correlated with oxidative stress. Smoking cessation remains the most effective preventive strategy

Risk of Bias Assessment

Table 2 presents the risk of bias assessment for the included studies, using appropriate tools according to study design, namely, the RoB 2 tool for randomized controlled trials and the ROBINS-I tool for nonrandomized or observational designs. Among the randomized trials, several demonstrated a low risk of bias, owing to adequate randomization procedures, blinding of participants and outcome assessors, and comprehensive outcome reporting. These studies adhered closely to methodological rigor, enhancing the reliability of their findings. A few trials had “some concerns,” primarily related to unclear blinding of outcome assessors or incomplete handling of missing data. In contrast, most of the nonrandomized or quasi-experimental studies were rated as having a moderate risk of bias. Common issues included lack of randomization, potential confounding due to absence of control groups, small sample sizes, and subjective exposure classifications, such as self-reported smoking cessation. Nevertheless, the majority of these studies measured biomarkers objectively, and several implemented repeated follow-ups or pre-/post-comparisons to strengthen internal validity. Overall, while a few studies raised methodological concerns, the collective evidence base was deemed sufficiently robust for drawing meaningful conclusions about the physiological effects of smoking cessation.

**Table 2:** Risk of bias assessment of included studies evaluating biomarker changes following smoking cessation RCT: randomized controlled trial; RoB 2: Revised Cochrane Risk of Bias Tool for Randomized Trials; ROBINS-I: Risk of Bias in Nonrandomized Studies-of Interventions; MI: myocardial infarction; DART: Diet and Reinfarction Trial

Study (author, year)	Study (author, year)	Study design	Tool applied	Risk of bias assessment
Korhonen et al., 2011 [13]	Korhonen et al., 2011	RCT (2 × 2 factorial)	RoB 2	Some concerns: randomization process well-described, but outcome assessors possibly not blinded; missing outcome data not clearly addressed
Stein et al., 2002 [14]	Stein et al., 2002	Clinical trial (nonrandomized)	ROBINS-I	Moderate: risk of confounding due to nonrandomization; no clear control over smoking reduction intensity; outcome assessment might be objective
Reichert et al., 2009 [15]	Reichert et al., 2009	Pilot clinical trial (pre-post design)	ROBINS-I	Moderate: small sample size and no control group raise concerns, but outcomes were objectively measured with repeated follow-up to support internal validity
Haustein et al., 2002 [16]	Haustein et al., 2002	Open-label clinical trial (quasi-randomized)	ROBINS-I	Moderate: nonrandom assignment to intervention/control; outcomes were objective, but allocation bias is possible
Haziza et al., 2020 [17]	Haziza et al., 2020	RCT (3-arm, parallel)	RoB 2	Low: adequate randomization, blinding, and comprehensive outcome reporting; robust methodology
Tonstad et al., 2005 [18]	Tonstad et al., 2005	RCT	RoB 2	Low: well-conducted trial with detailed outcome assessment and follow-up; low attrition risk
Chehne et al., 2002 [19]	Chehne et al., 2002	Observational (prospective pre/post)	ROBINS-I	Moderate: lack of randomization and control for confounding, but biomarker outcomes were objectively measured, and changes were time-bound and reproducible
Mah et al., 2013 [20]	Mah et al., 2013	RCT (placebo-controlled, double-blind)	RoB 2	Low: well-randomized, placebo-controlled, and blinded; outcomes clearly measured and reported.
Burr et al., 1992 [21]	Burr et al., 1992	Cohort (post-MI analysis from DART trial)	ROBINS-I	Moderate: observational component from a larger trial; self-reported smoking cessation could bias outcome classification
Dietrich et al., 2002 [22]	Dietrich et al., 2002	RCT (double-blind, placebo-controlled)	RoB 2	Low: strong methodology, blinding, and appropriate measurement of biomarkers; good reporting

Discussion

Overall Biomarker Recovery

The findings of this review suggest that smoking cessation is associated with measurable improvements across several biological pathways implicated in cardiovascular risk. Inflammatory biomarkers, including high-sensitivity CRP, TNF-α, IL-6, and fibrinogen, generally declined within the first several weeks following cessation. Markers of oxidative stress, particularly 8-iso-prostaglandin F2α and F2-isoprostanes, demonstrated some of the earliest improvements, suggesting attenuation of lipid peroxidation and oxidative vascular injury shortly after smoking abstinence. Favorable changes were also reported in lipid-related parameters, including total cholesterol, LDL cholesterol, and cholesterol ratios, alongside reductions in WBC count and other hematologic indices. Functional vascular measures, including flow-mediated dilation and maximal VO₂, also improved in some studies. Taken together, these findings are consistent with a pattern of gradual and partial biologic recovery across inflammatory, oxidative, metabolic, and vascular domains, although the extent and durability of these changes remain uncertain because of heterogeneity among the included studies and the predominance of nonrandomized evidence.

Temporal Patterns of Inflammatory and Oxidative Recovery

Inflammatory and oxidative stress biomarkers exhibited distinct but partially overlapping recovery trajectories following smoking cessation. Acute-phase inflammatory mediators such as TNF-α and soluble TNF receptors declined within approximately six to seven weeks in some cohorts [15], while reductions in high-sensitivity CRP were observed in studies with follow-up durations extending to 12 weeks [13] and 90 days [17]. Endothelial adhesion molecules appeared to respond more gradually, as reductions in intercellular adhesion molecule-1 (ICAM-1) were most evident among participants who combined smoking cessation with broader lifestyle modification strategies [18]. In contrast, oxidative stress markers demonstrated more rapid responsiveness to smoking abstinence. Chehne et al. reported substantial reductions in 8-epi-prostaglandin F2α within one to two weeks after cessation, with biomarker levels increasing following smoking relapse [19]. Similarly, Mah et al. demonstrated that seven days of smoking abstinence, particularly when combined with γ-tocopherol supplementation, was associated with reductions in MDA and improvement in endothelial function [20]. These findings suggest that oxidative stress pathways may respond rapidly to removal of tobacco exposure, whereas inflammatory and endothelial processes may normalize over a comparatively longer timeframe.

Lipid and Hematologic Recovery

Lipid and hematologic biomarkers generally improved over a longer post-cessation timeframe. Total cholesterol, LDL cholesterol, and the cholesterol ratio declined over 12 weeks in the study by Korhonen et al. [13], while Tonstad et al. demonstrated sustained reductions in oxidized LDL over eight months [18]. Fibrinogen levels also decreased within 26 weeks in Haustein et al. [16] and were associated with improved post-myocardial infarction outcomes in the Diet and Reinfarction Trial (DART) cohort [21]. WBC counts declined in parallel with fibrinogen, supporting the close relationship between inflammatory and thrombotic pathways in smoking-related cardiovascular injury. BMI appeared to influence biomarker recovery, as participants with higher BMI demonstrated greater reductions in F2-isoprostanes following antioxidant supplementation [22], whereas normal-weight individuals in Haziza et al. exhibited broader biomarker improvement after switching to a heated tobacco system [17]. Sex-specific differences were evaluated less consistently, although female-only cohorts demonstrated biomarker trajectories comparable to those observed in mixed populations [13,15]. Overall, the differing rates of recovery across biomarker classes suggest a sequential pattern of physiologic normalization in which oxidative stress improves earliest, followed by inflammatory, lipid, and hematologic recovery.

Mechanistic Interpretation

The observed biomarker trajectories are biologically consistent with established mechanisms of smoking-related vascular injury. Cigarette smoke promotes oxidative stress, leukocyte activation, endothelial dysfunction, and cytokine-mediated inflammation, all of which contribute to atherogenesis and thrombosis. Reductions in WBC count following smoking cessation may reflect attenuation of chronic inflammatory stimulation and normalization of leukocyte turnover [23]. Similarly, rapid declines in F2-isoprostanes and related oxidative stress markers likely correspond to reduced reactive oxygen species generation and diminished lipid peroxidation after removal of tobacco exposure [19,20]. Improvements in lipid-associated biomarkers may reflect gradual restoration of endothelial and lipoprotein function, including recovery of HDL-mediated anti-inflammatory activity [24]. Collectively, these findings support a biologically plausible sequence in which attenuation of oxidative stress precedes inflammatory downregulation and subsequent improvement in lipid and hematologic profiles, contributing to progressive cardiovascular risk reduction [25].

Study Contributions and Research Gaps

This review contributes to the current literature by synthesizing the temporal patterns of biomarker recovery following smoking cessation across multiple cardiovascular pathways. Oxidative stress markers appeared to improve within one to two weeks, inflammatory mediators within several weeks, and lipid or hemorheologic parameters over a comparatively longer period [26]. The review also identifies biomarker subsets that may be particularly responsive to smoking cessation, including F2-isoprostanes, TNF-α, and fibrinogen, while highlighting potential modifiers of recovery such as BMI and adjunctive interventions [27,28]. Although sex-related differences appeared limited in the available evidence, endothelial and oxidative stress responses remain insufficiently characterized in sex-stratified analyses. Collectively, these findings support the need for future studies incorporating longer follow-up periods, standardized biomarker assessment, and more individualized evaluation of metabolic and behavioral factors influencing post-cessation recovery [29].

Limitations and Future Directions

Several methodological limitations should be considered when interpreting these findings. Although multiple studies demonstrated favorable biomarker changes after smoking cessation, a substantial proportion of the included evidence originated from nonrandomized or quasi-experimental designs that may be vulnerable to confounding and selection bias. Considerable heterogeneity was also present across cessation strategies, follow-up duration, biomarker selection, and analytical methodology. In addition, biochemical verification of smoking abstinence, such as exhaled carbon monoxide or cotinine testing, was not consistently performed across the included studies, and reliance on self-reported abstinence in some investigations may have introduced exposure misclassification. In particular, oxidative stress markers such as MDA were inconsistently reported, limiting cross-study comparability. Furthermore, most studies evaluated relatively short-term biomarker responses, with limited evidence regarding the durability of these physiologic changes beyond six months. Future research should prioritize rigorously designed prospective studies comparing different cessation approaches, using standardized biomarker panels, consistent biochemical verification of abstinence, and extended longitudinal follow-up. Integration of transcriptomic, proteomic, and metabolomic profiling may further improve understanding of individualized cardiovascular recovery after smoking cessation.

Clinical and Public Health Implications

The biomarker changes observed after smoking cessation may reflect early modulation of cardiovascular risk pathways before measurable long-term clinical outcome improvements become apparent. Reductions in inflammatory biomarkers, such as CRP and fibrinogen, alongside improvements in oxidative stress markers and lipid oxidation, suggest attenuation of endothelial and atherothrombotic injury following smoking abstinence. One included study evaluated switching from conventional cigarettes to a menthol tobacco heating system rather than complete smoking cessation; therefore, its findings should be interpreted separately as evidence of reduced combustible smoke exposure rather than full physiologic recovery after abstinence. Overall, the findings may provide clinically relevant physiologic markers that can support patient counseling and reinforce adherence to cessation interventions, while also supporting continued investment in smoking cessation programs and preventive cardiovascular strategies.

## Conclusions

This systematic review suggests that smoking cessation is associated with progressive recovery across multiple biological pathways implicated in cardiovascular risk. Oxidative stress markers, particularly F2-isoprostanes, demonstrated some of the earliest improvements following cessation, whereas inflammatory mediators, including CRP, TNF-α, and fibrinogen, generally declined over subsequent weeks. Lipid-related and hemorheologic parameters improved more gradually over several months. The findings further indicate that factors such as BMI and adjunctive interventions may influence the trajectory of biomarker normalization. Although methodological heterogeneity and limited long-term follow-up remain important limitations within the available literature, the current evidence supports smoking cessation as a key strategy for cardiovascular risk reduction and highlights the potential clinical value of biomarker-based assessment in monitoring early physiologic recovery after smoking abstinence.
